# Sensor based time budgets in commercial Dutch dairy herds vary over lactation cycles and within 24 hours

**DOI:** 10.1371/journal.pone.0264392

**Published:** 2022-02-25

**Authors:** P. R. Hut, S. E. M. Kuiper, M. Nielen, J. H. J. L. Hulsen, E. N. Stassen, M. M. Hostens

**Affiliations:** 1 Department Population Health Sciences, Division Farm Animal Health, Faculty of Veterinary Medicine, Utrecht University, Utrecht, The Netherlands; 2 Vetvice/Cowsignals, Bergen op Zoom, The Netherlands; 3 Adaptation Physiology Group, Department of Animal Sciences, Wageningen University & Research, Wageningen, The Netherlands; 4 Department of Reproduction, Obstetrics and Herd Health, Ghent University, Merelbeke, Belgium; Michigan State University, UNITED STATES

## Abstract

Cows from 8 commercial Dutch dairy farms were equipped with 2 sensors to study their complete time budgets of eating, rumination, lying, standing and walking times as derived from a neck and a leg sensor. Daily sensor data of 1074 cows with 3201 lactations was used from 1 month prepartum until 10 months postpartum. Farms provided data over a 5 year period. The final models (lactational time budget and 24h time budget) showed significant effects of parity, farm and calving season. When primiparous cows were introduced in the lactational herd, they showed a decrease in lying time of 215 min (95% CI: 187–242) and an increase in standing time of 159 min (95% CI: 138–179), walking time of 23 min (95% CI: 20–26) and rumination time of 69 min (95% CI: 57–82). Eating time in primiparous cows increased from 1 month prepartum until 9 months in lactation with 88 min (95% CI: 76–101) and then remained stable until the end of lactation. Parity 2 and parity 3+ cows decreased in eating time by 30 min (95% CI: 20–40) and 26 min (95% CI: 18–33), respectively, from 1 month before to 1 month after calving. Until month 6, eating time increased 11 min (95% CI: 1–22) for parity 2, and 24 min (95% CI: 16–32) for parity 3+. From 1 month before calving to 1 month after calving, they showed an increase in ruminating of 17 min (95% CI: 6–28) and 28 min (95% CI: 21–35), an increase in standing time of 117 min (95% CI: 100–135) and 133 min (95% CI: 121–146), while lying time decreased with 113 min (95% CI: 91–136) and 130 min (95% CI: 114–146), for parity 2 and 3+, respectively. After month 1 in milk to the end of lactation, lying time increased 67 min (95% CI: 49–85) for parity 2, and 77 min (95% CI: 53–100) for parity 3+. Lactational time budget patterns are comparable between all 8 farms, but cows on conventional milking system (CMS) farms with pasture access appear to show higher standing and walking time, and spent less time lying compared to cows on automatic milking system (AMS) farms without pasture access. Every behavioral parameter presented a 24h pattern. Cows eat, stand and walk during the day and lie down and ruminate during the night. Daily patterns in time budgets on all farms are comparable except for walking time. During the day, cows on CMS farms with pasture access spent more time walking than cows on AMS farms without pasture access. The average 24h pattern between parities is comparable, but primiparous cows spent more time walking during daytime compared to older cows. These results indicate a specific behavioral pattern per parameter from the last month prepartum until 10 months postpartum with different patterns between parities but comparable patterns across farms. Furthermore, cows appear to have a circadian rhythm with varying time budgets in the transition period and during lactation.

## Introduction

Continuous monitoring of dairy cattle with sensor technology provides opportunities to detect deviations based on rolling averages (i.e. heat detection) and a better understanding of the behavior of these animals [1–3]. As sensor technology develops, it is also becoming possible to use sensor data for disease detection [4–7], for disease and fertility prediction [8, 9], assessment of welfare [10] and decision support in management [11].

Although much research is focused on early detection of disease, some also elucidate zootechnical influences on specific behavior [12–14]. For example, dairy cow behavior differs between farms with automatic milking systems (AMS) and conventional milking systems (CMS) [15]. It also varies with group size and stocking density [16, 17] and with pasture access compared to indoor housing [12]. In addition to zootechnical aspects, specific cow attributes present behavioral differences. Behavioral patterns of dairy cows differ in the dry period compared to the lactational period. Further, primiparous cows behave differently compared to multiparous cows [9, 18].

Combining several behavioral parameters can lead to a better understanding of the dairy cows’ time budget [19, 20]. For instance, when cows are lying down rumination time is higher compared to rumination time when cows are not. When cows spent more time ruminating, these periods were associated with less dry matter intake (DMI), indicating that cows do not eat and ruminate at the same time [21]. Time budgets can be approached as a combined set of several behavioral parameters per day and over a certain period in time, such as 24h patterns. For example, dairy cows show a diurnal pattern in feed intake depending on milking time and fresh feed delivery [22]. They also seem to have a circadian pattern, based on individual cow positions [23]. Moreover, they exhibit changes in circadian rhythms that can be used to detect estrus and disease [24].

While others have studied dairy cattle using extensive sensor data, these studies reported only one or two behavioral parameters as time budgets [9, 18, 25]. Thus, complete time budgets combining data for feeding behavior (eating time and rumination time), lying behavior and walking behavior (standing time and walking time) seem lacking. This is also true of behavioral profiles based on 24h patterns and studies based on sensor data originating from commercial dairy farms.

The goal of this retrospective observational study is to combine sensor data from 2 types of sensors (3-dimensional neck and leg accelerometers) to create a complete time budget of dairy cows throughout the lactation cycle, to gain a better understanding of dairy cow behavior and sensor data in a commercial setting while correcting for parity, milking type and calving season taken into account. In addition, the combined daily sensor data creates a time budget of the daily behavioral pattern allowing the creation of 24h patterns. This reveals the effects of parity, months in lactation and differences between farms, extending previous reports which are mostly studied on a single farm, allowing comparisons between time budgets among farms. The results of this study could provide a benchmark for different dairy farming systems.

## Materials and methods

### Farms, animals and sensors

**Fig 1:**
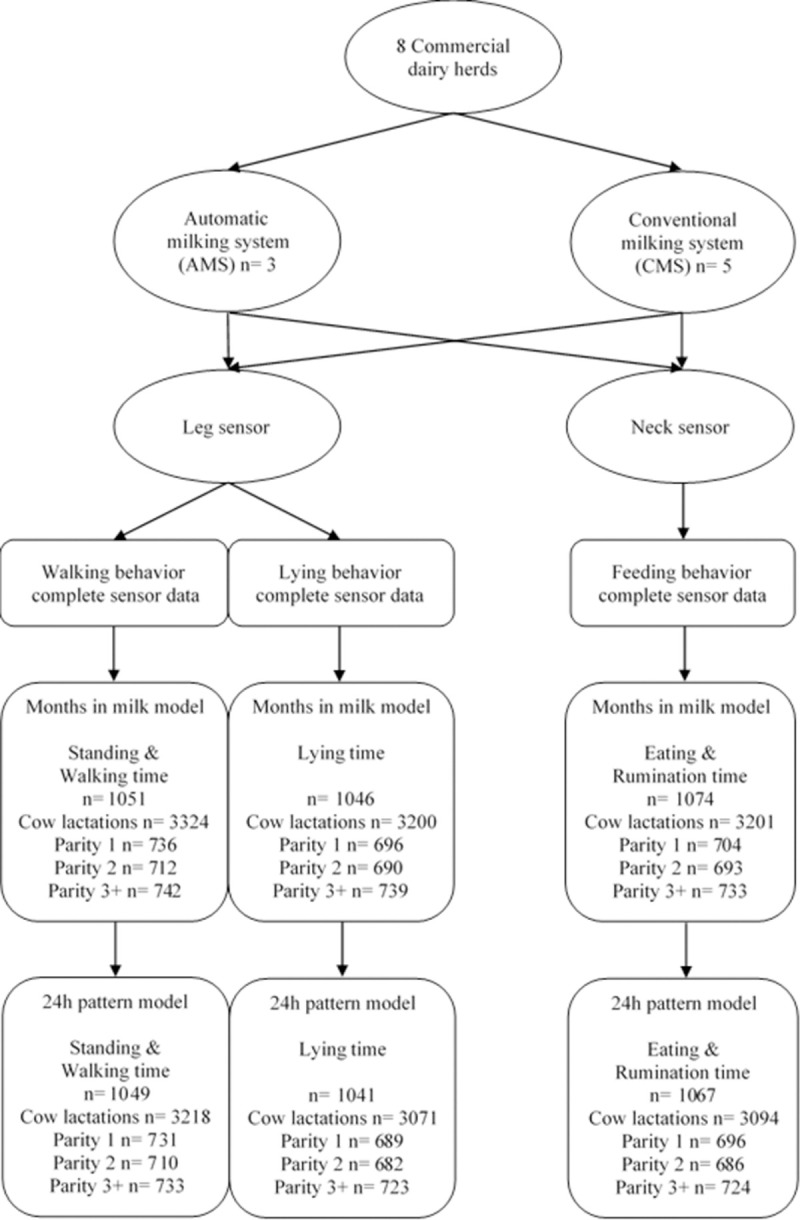
Distribution of all cows used in this study equipped with both leg and neck sensors per type of farm (AMS = automatic milking system, CMS = conventional milking system) per statistical model (months in milk model and 24h pattern model).

All dairy cows on the 8 commercial dairy farms in The Netherlands included in this study were equipped with 2 types of sensors. Details of these farms, with an average herd size of 140 cows, are described in Table 1 and in our previous publication on lameness [26]. To monitor feeding behavior (eating time and rumination time), commercially available “Nedap Smarttag Neck” sensors (Nedap, Groenlo, The Netherlands) were attached to the neck collar of each cow and the commercially available “Nedap Smarttag Leg” sensors were attached to one of the front legs of each cow, to monitor walking (walking time and standing time) and lying behavior (lying time). Both sensors were validated by previous studies and have high correlations between observed and reported behavioral parameters (0.88–0.97) [27–29]. In total, 1074 cows with 3201 lactations were available in this study. The use of such sensors in a commercial dairy herd is not considered an animal experiment under Dutch law, hence no formal ethical approval was needed (see also [26]). The number of cows per sensor based behavioral parameter is presented in Fig 1. For visualization purposes, farms were grouped by type of milking system (AMS, N = 3 / CMS, N = 5) where cows on CMS farms also had pasture access during parts of spring, summer and autumn for at least 120 days/year for at least 6h/day as a part of a subsidized Dutch system to stimulate pasture access for dairy cows. This resulted in 2 groups: the AMS-C (automatic milking system–confined) group and the CMS-P (conventional milking system–pasture access) group.

**Table 1:** Characteristics of 8 commercial dairy farms in The Netherlands used in this retrospective observational study.

Farm	Herd size	DP cubicle bedding far off	DP cubicle/ yard bedding close up	Average DP length (25%-75% IQR)	Cubicle bedding lactation	Milking system	Pasture access	Production level (kg milk/cow/year)
**1**	170	Deep litter	Straw yard	41 (31–46)	Deep litter	AMS	No	10786
**2**	130	Deep litter	Straw yard	39 (30–41)	Deep litter	AMS	No	11177
**3**	110	Mattress	Mattress	45 (40–51)	Mattress	AMS	No	9341
**4**	110	Mattress	Straw yard	39 (33–43)	Mattress	CMS	Yes	9314
**5**	140	Deep litter	Deep litter	35 (30–40)	Deep litter	CMS	Yes	9256
**6**	170	Mattress	Mattress	37 (32–42)	Mattress	CMS	Yes	9243
**7**	175	Deep litter	Straw yard	42 (32–48)	Deep litter	CMS	Yes	9109
**8**	120	Mattress	Mattress	45 (37–49)	Mattress	CMS	Yes	9197

AMS = automatic milking system. CMS = conventional milking system. DP = dry period. IQR = interquartile range. Deep litter is related to cubicle systems where a straw yard means a free-range area. Pasture access is for lactating animals only.

### Study design

For this retrospective observational study, sensor data of 1074 cows with 3201 lactations was collected over a 5-year period from January 1^st^ 2016 until December 31^st^ 2020. Sensor data was provided by Nedap Livestock Management (Nedap, Groenlo, The Netherlands). Behavioral parameter sensor data was collected in two different formats in minutes per 2 hours (min/2h) and minutes per 24h (min/24h) data files (CSV). Sensor data per animal was aligned around the day of calving.

The data files with daily summations (min/24h) were averaged per 30 days, creating “months” before and after calving. The day before calving, calving day and the day after calving were used separately as “month 0” because of specific alterations in the behavioral patterns on these days around calving [9]. Month -1 consisted of d -31 until d -2. Month 1 in lactation consisted of d 2 until d 31. Every month in lactation until month 10 consisted of 30-day cycles and do not represent calendar months. The data files with data per 2 hours were used to study the 24h pattern. The numbers of animals differ slightly between models due to sensor data transfer.

### Statistical analysis

To be able to analyze these data sets (over 150Gb), analysis was carried out using R via the Google Colab system, including packages: “car” [30], “carData” [31], “dplyr” [32], “emmeans” [33], “ggplot2” [34], “gridExtra” [35], “lme4” [36], “lmerTest” [37], “lsmeans” [38], “multcomp” [39], “multcompView” [40], “mvtnorm” [41], “plyr” [42], “readr” [43], “TH.data” [44], “tidyr” [45], and the R Project [46].

All statistical analyses including code scripts can be downloaded at https://github.com/Bovi-analytics/hut-et-al-2021. Independent variables used were the unique herd and animal identifier, parity of the animal(1, 2, 3+), months in milk (-1 to 10 for monthly analysis, 1–10 for 24h pattern in lactation to exclude dry period effects), calving season (spring: April/May/June, summer: July/August/September, autumn: October/November/December, winter: January/February/March), and 2 hour blocks (12 blocks from 0 to 22 for the 24h pattern) For visualization purposes, farms were divided in 2 groups: AMS-C (automatic milking system–confined) and CMS-P (conventional milking system–pasture access). A continuous ‘months in milk’ variable was also added as repeated measures to account for covariance over time. Separate models were built for each of the sensor values: eating time, rumination time, lying time, standing time and walking time. All final model residuals were checked for normal distribution with QQ plots.

First, all explanatory variables were tested in univariable linear mixed effect models taking into account a random effect of each animal nested within the fixed effect of the herd. Each of the univariable models showed a lower Akaike’s Information Criterion (AIC) compared to the null model only taking into account the random effect. Multivariable model building was based on AIC. First, a multivariable model was created with every factor in a complete model. Second, possible pairwise interactions were created between all offered variables. Two final multilevel models were created based on the lowest AIC: one final model per behavioral parameter for the complete daily time budget and a second final model for the 24h pattern. These models had the lowest AIC in every behavioral parameter analysis. The final model (model 1) for time budgets over lactation cycles as independent variable resulted in the following model: months in milk, parity, farm and calving season were used as fixed effects taking into account a repeated effect of months in milk nested within each cow. Biologically relevant interactions were included, namely months in milk with parity, months in milk with farm and months in milk with calving season. The final model (model 2) for the 24h pattern based on 2 hourly sensor data was as follows: model 1 with 2h block as extra fixed effect and interactions between 2h block and parity, 2h block and farm, and 2h block and calving season considering a repeated effect of months in milk nested within each cow. Final model effects were reported and plotted as least square means (LSM) with 95% confidence intervals (95% CI). Multiple comparisons contrasts were adjusted using the Tukey method. Per graph, significant differences (P<0.05) were present when the 95% CI error bars did not overlap.

## Results

### Monthly time budget models

**Fig 2:**
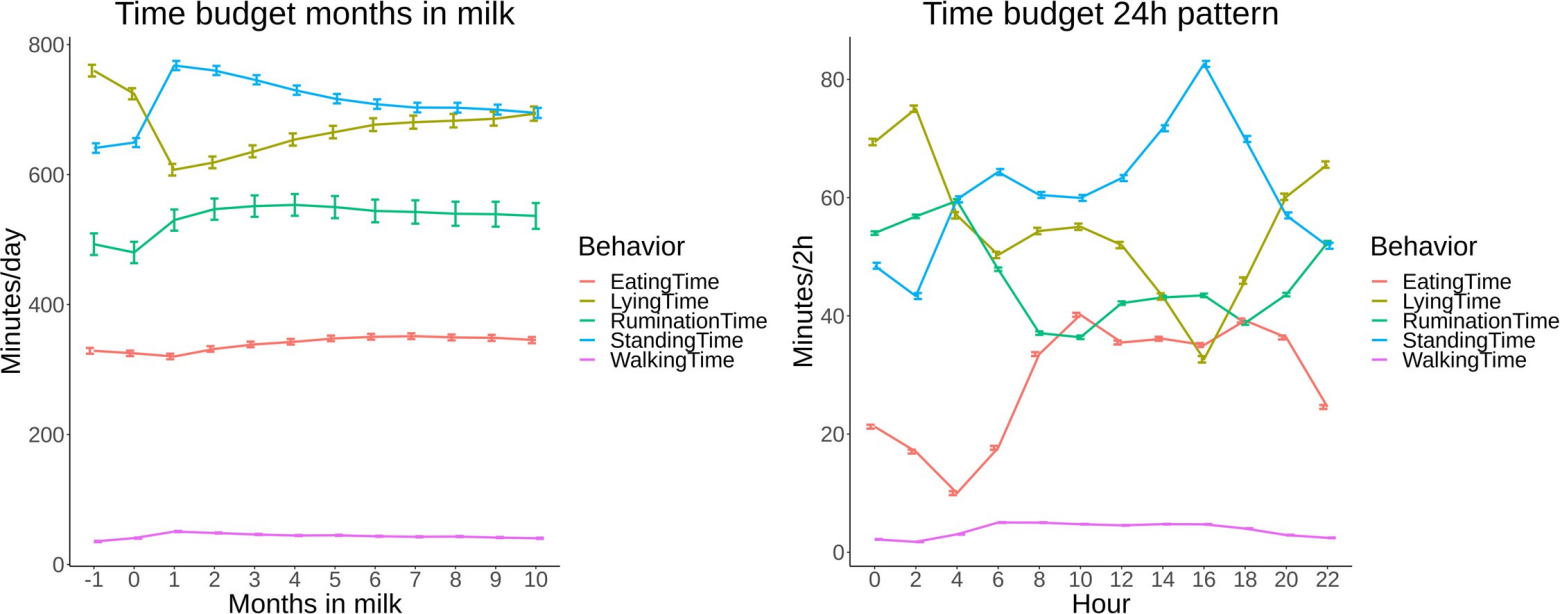
A,B: Overall time budget (eating, rumination, lying, walking and standing) based on least square means (LSM) with 95% confidence intervals (95% CI) of all cows on 8 commercial dairy farms in The Netherlands from 1 month before calving until 10 months in milk with “month 0” consisting of d-1, d0 and d+1 (A) and their overall 24h pattern time budget (B).

The complete time budget of all cows in this study from 1 month before calving until 10 months in milk is presented in Fig 2A. These overall estimates show that behavioral parameters have a pattern during the lactational cycle. Lying time decreased from 1 month before calving until 1 month after calving. After, lying time gradually increased towards the end of lactation. Standing time showed an inverse pattern of lying time. While eating time decreased after calving, rumination time increased up to 4 months in milk. Eating time increased after 1 month in milk towards 6 months in milk and seemed to decrease afterwards. Walking time increased the first month after calving and decreased afterwards. In total, daily time budgets changed over the course of lactation with most notable changes from 1 month before until 1 month after calving when cows transitioned from the dry period into the lactational period.

**Fig 3:**
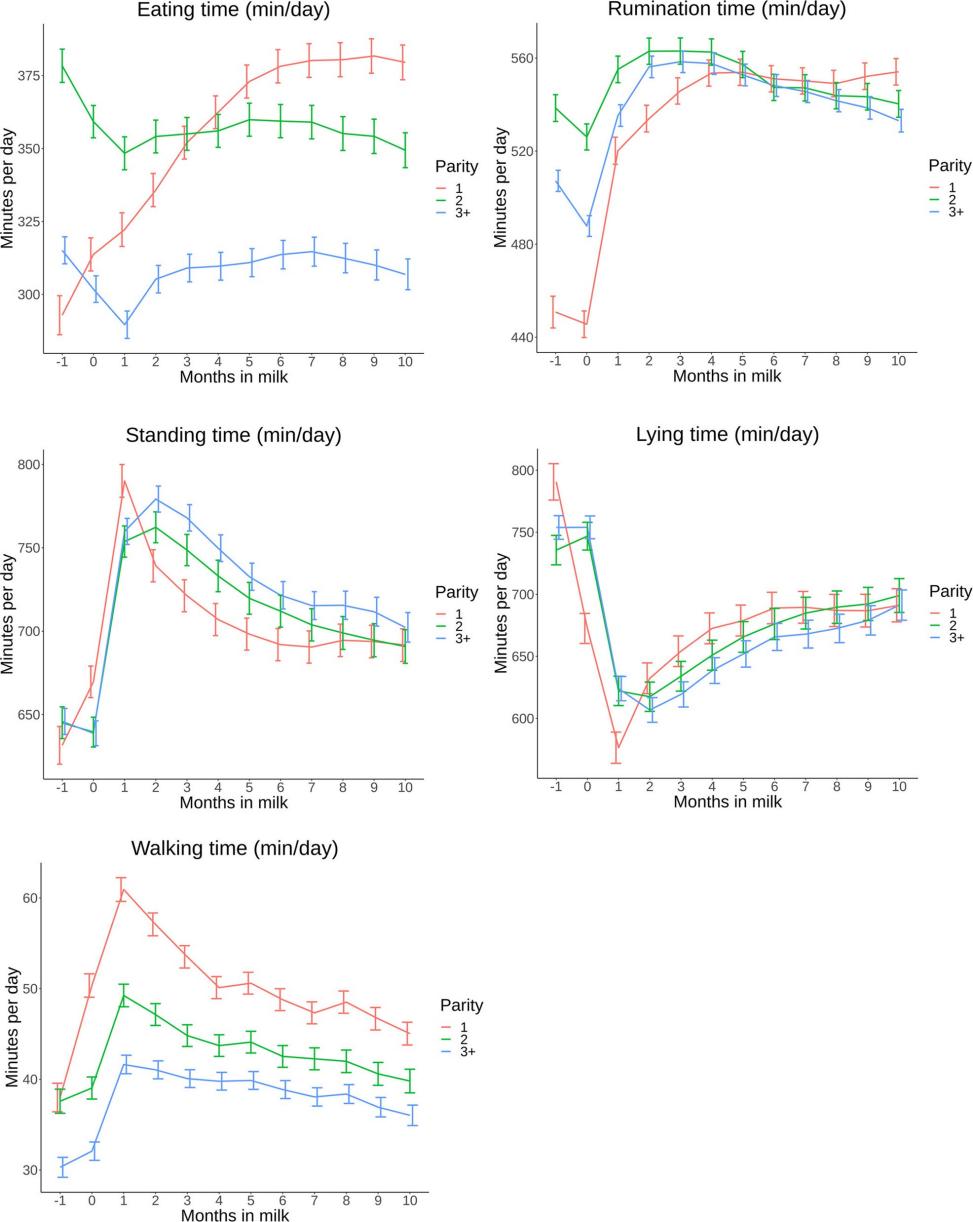
A,B,C,D,E: Time budget parameters in minutes per day (min/day) based on least square means (LSM) with 95% confidence intervals (95% CI) grouped by parity (1, 2 and 3+) on 8 commercial dairy farms in The Netherlands from 1 month before calving until 10 months in milk with “month 0” consisting of d-1, d0 and d+1.

**Fig 4:**
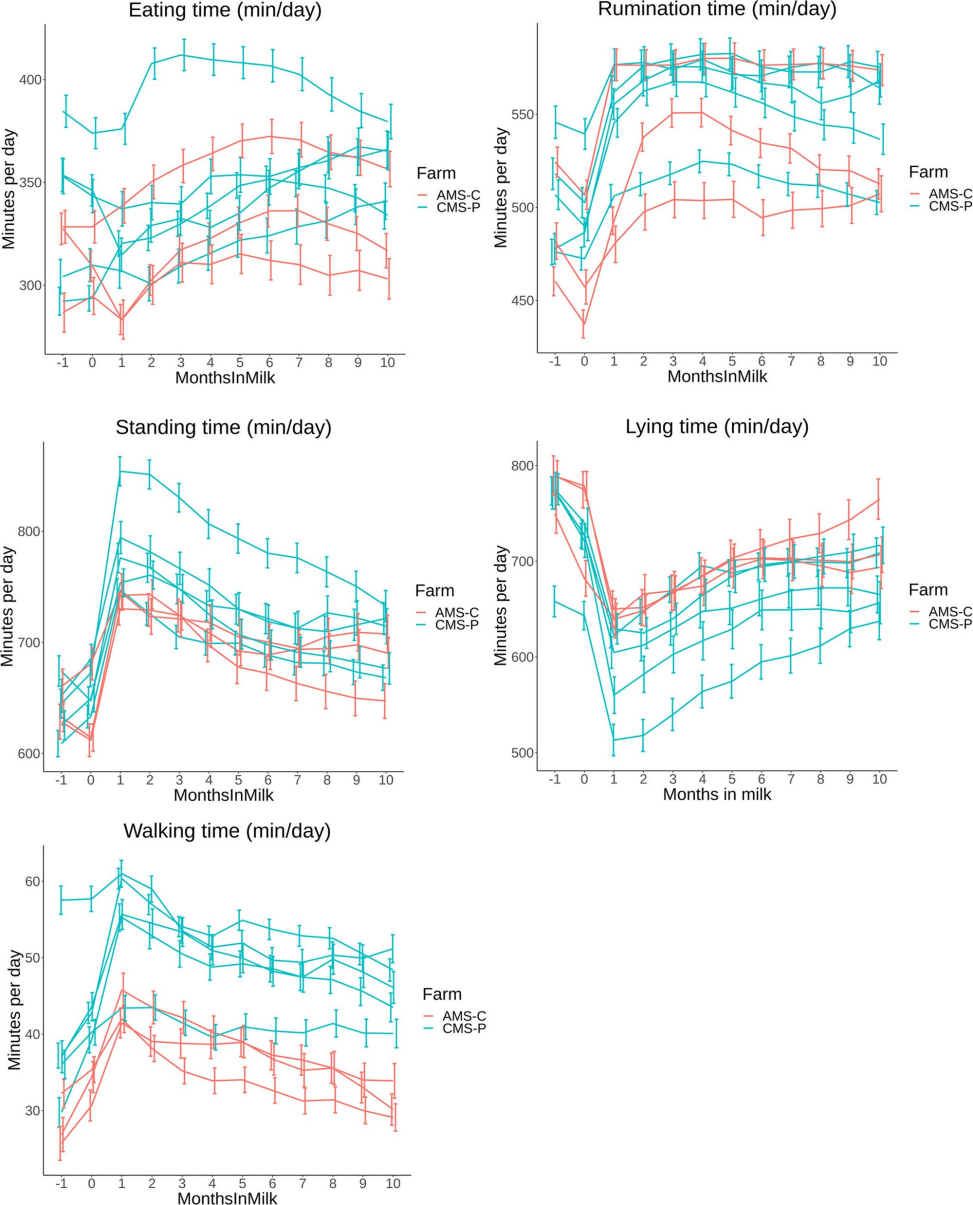
A,B,C,D,E: Time budget parameters in minutes per day (min/day) based on least square means (LSM) with 95% confidence intervals (95% CI) per farm grouped by color: red = AMS-C, blue = CMS-P (automatic milking system–confined, and conventional milking system–pasture access) on 8 commercial dairy farms in The Netherlands from 1 month before calving until 10 months in milk with “month 0” consisting of d-1, d0 and d+1.

**Fig 5:**
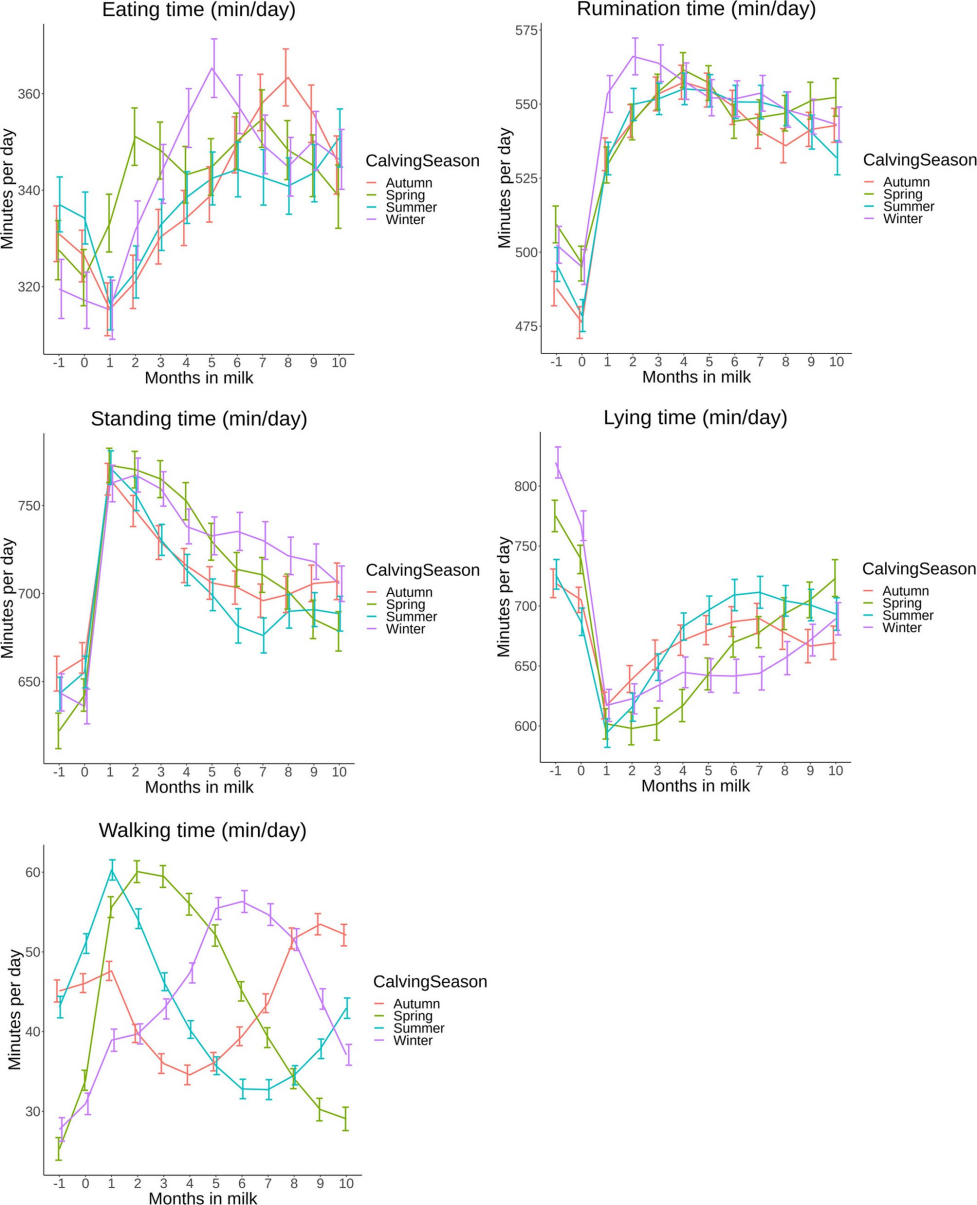
A,B,C,D,E: Time budget parameters in minutes per day (min/day) based on least square means (LSM) with 95% confidence intervals (95% CI) grouped by calving season (Spring, Summer, Autumn, Winter) on 8 commercial dairy farms in The Netherlands from 1 month before calving until 10 months in milk with “month 0” consisting of d-1, d0 and d+1.

The final model showed significant effects (P<0.001) of parity, farm and calving season. Therefore, the LSM and 95% CI predictions per behavioral parameter for parity groups (1, 2 and 3+), farms and calving season are presented in Figs 3–5, all exact estimates are available on the previously reported open access repository.

Eating time of primiparous cows increased with 88 min (95% CI: 76–101) (Fig 3A) from 1 month before first calving until month 9 in milk. Cows in parity group 2 and 3+ spent more time eating (30 min (95% CI: 20–40) and 26 min (95% CI: 18–33)) the first month pre partum compared to 1 month post partum. After the first month, eating time for parity 2 and 3+ increased until 6 months in milk (11 min (95% CI: 1–21) and 24 min (95% CI: 16–32), respectively). Eating time differed between parity groups over the presented period, except for month 3 and for in milk for parity 1 and 2.

For rumination time, primiparous cows had an increase of 69 min (95%CI: 57–82) (Fig 3B) from 1 month before calving to 1 month after calving. A further incline of 33 min (95% CI: 22–44) up to 4 months in milk was present and remained more or less stable during the rest of lactation. Multiparous cows showed an increase of between 17 min (95% CI: 6–28) and 28 min (95% CI: 21–35) from 1 month pre partum to 1 month post partum for parity 2 and 3+, respectively. Towards peak lactation a further increase of 8 min (95% CI: 3–18) for parity 2 and 23 min (95% CI: 15–31) for parity 3+ was present, followed by a decline of around 20 min until the end of lactation for both parity groups.

Standing time of primiparous cows had a large increase (Fig 3C) of 159 min (95% CI: 138–179) between 1 month before calving and 1 month after calving. In month 2 post partum, their standing time decreased by 51 min (95% CI: 33–69), with a further decrease of 48 min (95% CI: 30–66) over the remainder of lactation. Multiparous cows showed a slightly different pattern. Their standing time increased with 117 min (95% CI: 100–135) for parity 2 and with 133 min (95% CI: 121–146) for parity 3+. Towards the end of lactation, standing time decreased with 72 min (95% CI: 53–90) for parity 2 and with 77 min (95% CI: 63–91) for parity 3+.

A large decrease in lying time of 215 min (95% CI: 187–242) was shown by primiparous cows (Fig 3D) from 1 month before calving to 1 month after calving. Their lying time increased by 113 min (95% CI: 89–137) at around 7 months in milk. Older cows show a similar pattern but with less decline after calving (113 min (95% CI: 91–136) for parity 2 and 130 min (95% CI: 114–146) for parity 3+). From month 1 in milk to the end of lactation, multiparous cows increased in lying time with 67 min (95% CI: 49–85) and 77 min (95% CI: 53–100) for parity 2 and 3+, respectively.

The patterns of walking time (Fig 3E) were similar compared to standing time where primiparous cows experienced the largest increase in walking time between 1 month before and 1 month after calving (23 min, 95% CI: 20–26). Until month 10 in milk, walking time decreased with 16 min (95% CI: 13–19). The course for parity 2 and 3+ was similar in pattern. Walking time differed between parity groups over the presented period, except for month -1 for parity 1 and 2.

Differences between farms are illustrated in Fig 4 and grouped by color for farm types: AMS-C and CMS-P. Sensor data from the neck sensor (eating and rumination time) showed overlapping patterns between farms, without distinction between the two farm types. The sensor data from the leg sensor (standing, lying and walking time) presented distinction between the two farm types, with cows from AMS-P farms showing higher lying time and lower standing and walking time compared to CMS-P cows.

The effects of calving season on daily time budgets is shown in Fig 5. Cows calving in winter show a steeper incline in eating time after calving compared with cows calving in other seasons (Fig 5A). Other behavioral parameters showed similar patterns per season except for walking time. The effects of calving season (Fig 5E) show the effects of pasture access in spring, summer and the first part of autumn for CMS-P cows. These effects were analyzed separately with factor farm as random factor, results are available on the previously mentioned online open access repository.

### 24h time budget models

The daily time budget based on 2 hourly sensor data blocks is presented in Fig 2B. During daytime, cows spent most time eating, standing and walking, while lying and rumination occurred mostly during the night.

**Fig 6:**
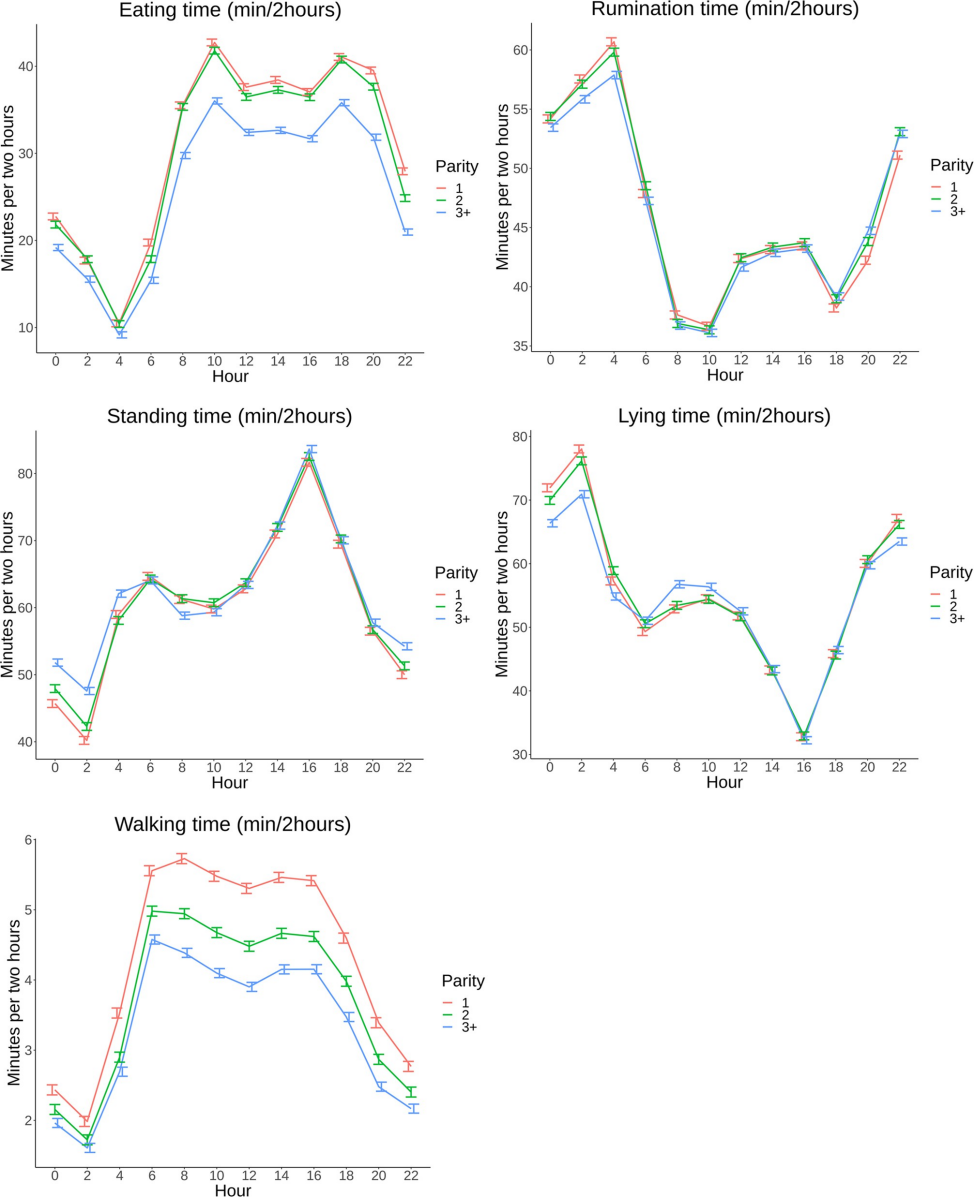
A,B,C,D,E: 24h pattern in minutes per 2 hours (min/2hours) based on least square means (LSM) with 95% confidence intervals (95% CI) grouped by parity: 1, 2 and 3+ on 8 commercial dairy farms in The Netherlands from 00:00AM until 22:00PM.

**Fig 7:**
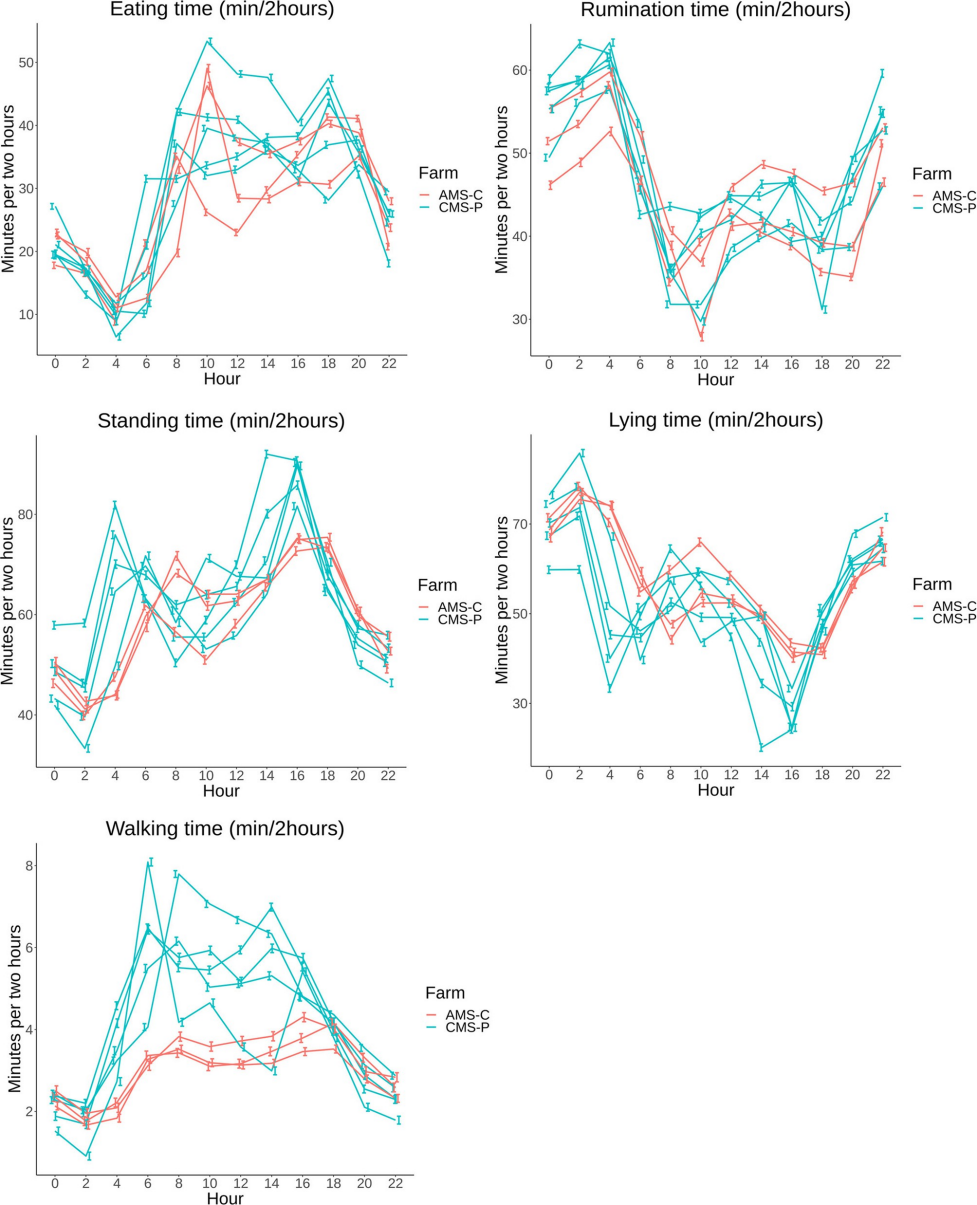
A,B,C,D,E: 24h pattern in minutes per 2 hours (min/2hours) based on least square means (LSM) with 95% confidence intervals (95% CI) per farm grouped by color: red = AMS-C, blue = CMS-P (automatic milking system–confined, and conventional milking system–pasture access) on 8 commercial dairy farms in The Netherlands from 00:00AM until 22:00PM.

**Fig 8:**
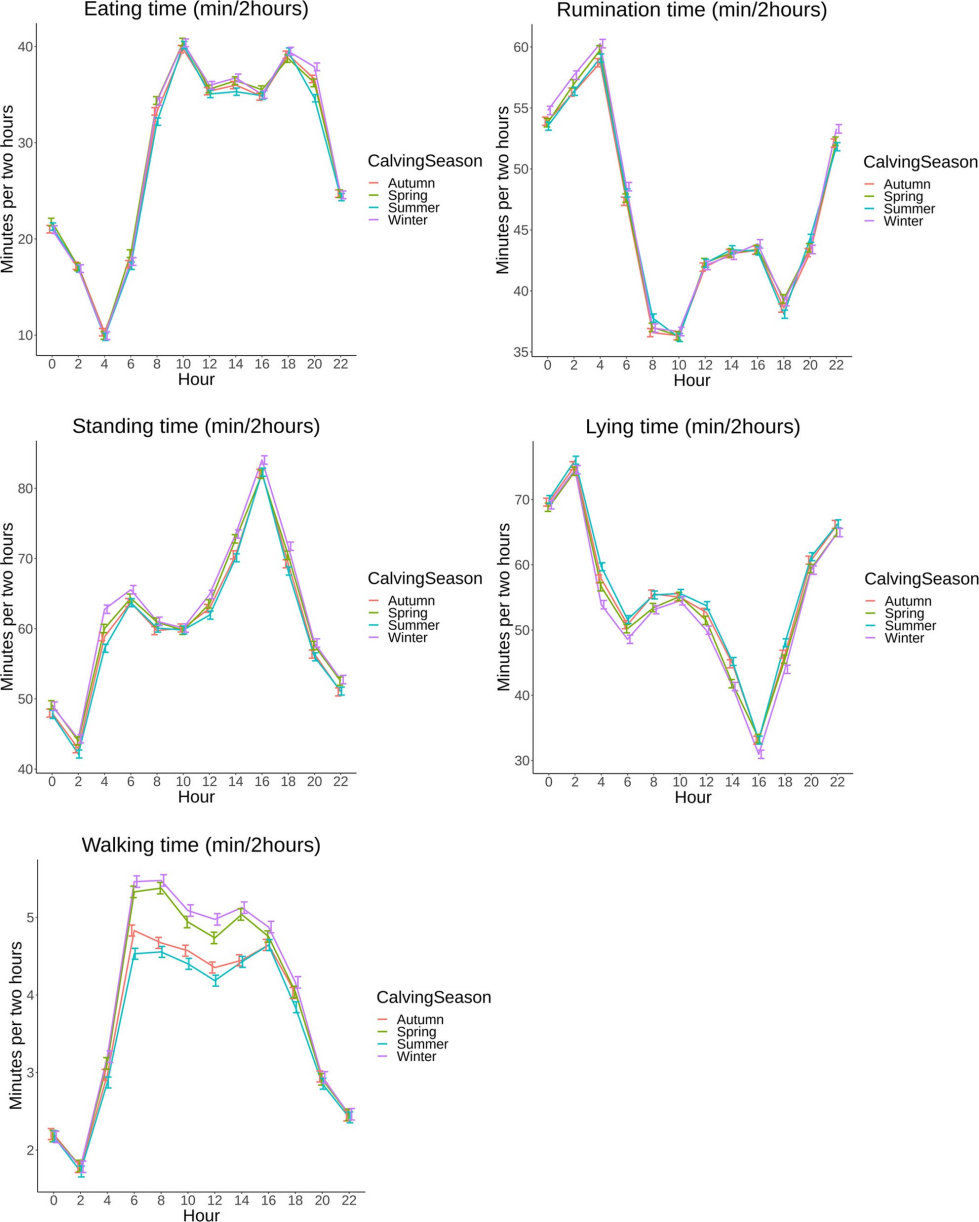
A,B,C,D,E: 24h pattern in minutes per 2 hours (min/2hours) based on least square means (LSM) with 95% confidence intervals (95% CI) grouped by calving season (Spring, Summer, Autumn, Winter) on 8 commercial dairy farms in The Netherlands from 00:00AM until 22:00PM.

The final model showed significant effects (P<0.001) of parity, farm and calving season. The LSM and 95% CI predictions per 24h pattern of each behavioral parameter for parity groups (1, 2 and 3+), farms and calving season are presented in Figs 6–8, all exact estimates are available on the previously reported open access repository.

Cows in parity group 3+ spent less time eating (32 min/2h (95% CI: 32–33)) during the entire 24h course compared to younger cows (38 min/2h (95% CI: 37–38) (Fig 6A), while rumination patterns (Fig 6B) are more or less comparable across parities. During the night, cows in parity group 3+ spent less time lying (Fig 6D) compared with the other groups (71 min/2h (95% CI: 70–72) versus 77 min/2h (95% CI: 75–79)), but lying time during the morning was higher (57 min/2h (95% CI: 56–58)) in the group of older cows compared to younger cows (53 min/2h (95% CI: 52–54)). Parity groups showed lower walking time (Fig 6E) with increasing parity during the 24h pattern with at noon, for example, 5.3 min/2h (95% CI: 5.2–5.4) for parity 1, 4.5 min/2h (95% CI: 4.4–4.6) for parity 2, and 3.9 min/2h (95% CI: 3.8–4.0).

The 24h patterns of the different farms were very similar with a daytime pattern of mainly eating (20–50 min/2h), standing (50–100 min/2h) and walking (3–8 min/2h) during the day while during the night rumination (45–65 min/2h) and lying (50–90 min/2h) were most dominant (Fig 7). Cows from AMS-C farms showed less walking time (3–4 min/2h) during the morning compared to cows from CMS-P farms (4–8 min/2h).

Daily patterns separated by calving season only showed differences for walking time: cows that calved in winter or spring showed higher activity during the daytime of 5.5 min/2h (95% CI: 5.4–5.6) versus 4.5 min/2h (95% CI: 4.4–4.6) for cows that calved in summer or autumn (Fig 8).

## Discussion

The goal of this study was twofold. First, we wanted to study how time budgets of dairy cows vary between the dry period and the lactational period, and vary over the lactation period. Second, we wanted to study the daily activity pattern. In this study we used sensor data of 1074 cows with 3201 cow lactations collected over a period of 5 years from 8 commercial Dutch dairy farms. The resultant sensor data of multiple parities per individual cow was modeled as cow nested within the fixed effect of the farm as a random factor and corrected for repeated observations over time. Our results show that primiparous cows present vastly different time budgets compared to multiparous cows, from 1 month before calving throughout lactation. Primiparous and multiparous cows present a distinct 24h pattern of lying and ruminating during the night and walking, standing and eating during the day.

Others have already studied sensor data in the transition period (from 3 weeks before until 3 weeks after calving) per day or week while we studied 1 month before and 1 month after calving [18, 25]. While our data shows comparable patterns as other studies of baseline behavioral sensor data output, we considered the last 31 days before calving, summed and averaged in min/day (and d-1, d0 and d+1 were studied separately as “month 0”). Because of the effect of calving on behavioral patterns, the day before calving, the day of calving and the day after calving were modeled as a separate “month” to prevent interference with baselines before and after calving [47–49]. Behavioral patterns in the transition period are subject to change [50]. Our data corroborated this, presenting differences between month -1 and month +1, even if our data includes healthy as well as less healthy cows. Others reported such transition patterns as useful parameters to relate to transition diseases [50–52].

Parity differences in behavioral parameters were described earlier, although mostly related to the transition period [9, 18, 53]. Where we only studied eating time, others studied meals per day, visits per meal, meal size, meal time, DMI and feeding rate. Younger cows spent more time eating with more meals, more visits, lower DMI, and lower feeding rate compared to older cows. Although that study was performed on 1 farm, our results on 8 farms for eating time are consistent with more eating time for primiparous cows compared to older cows [54]. Additionally, primiparous cows showed smaller bite size compared to multiparous cows, which could explain higher eating time with less DMI [55]. Primiparous cows also showed improved health and production when housed in a separate group the first month after calving [56]. All cows have energy requirements for milk production, but primiparous cows differ metabolically because they need energy for growth as well [57]. This suggests that the 24h patterns of primiparous cows were revealing more eating time and longer walking time patterns as the quantified effect of hierarchical differences between primiparous and multiparous cows. Primiparous cows also have less weight than older animals which might result in evasive behavior when conflicts for feed, milking order or resting places arise especially after introduction to the milking herd for the first time [58–60]. Combining these effects on behavior, health, production and growth, it could be advisable to house primiparous cows separate from multiparous cows, which is relatively simple to implement in larger herds [61].

While others have studied the effect of different housing and milking systems on lying behavior, daily behavioral patterns are complex and dynamic combinations of zootechnical circumstances, stocking density, ration and management [14, 62, 63]. For lying time the same trajectory was seen by others who report a drop in the early post partum period and a gradual rise towards the end of lactation [64]. The only behavioral parameter that follows the lactation curve is rumination indicating that peak production correlates with peak rumination and does not seem to coincide with peak lying time.

Our results suggest that differences among farms were associated with the management type. We suggest that these differences were most likely influenced by pasture access on CMS farms. However, when separating for calving season, the pasture access effect on walking time was especially strong for winter and spring calving cows. Previous studies have shown conflicting results regarding the effects of pasture access on behavior. Some report that cows on pasture have higher lying times [65] where others show lower lying, standing, and rumination times and higher eating time [66]. Our results show lower lying time and higher standing and walking time on farms with pasture access. Less lying and more standing time in our study could indicate higher waiting times before milking on CMS-P farms compared to AMS-C farms [20]. Also, other farm management differences as cubicle size and cubicle bedding could confound these results. On eating time, it probably takes more time to ingest the same amount of dry matter while grazing compared to complete ration feeding indoors. On AMS farms, cows have a more continuous flow of eating compared to farms with grazing [15]. However, eating and rumination times between AMS-C and CMS-P farms overlapped greatly. All farms fed a PMR (partial mixed ration) which typically contained 75% grass silage, 25% maize silage supplemented with different protein sources and balanced concentrates. In the CMS-P group, cows also had pasture access as part of the feeding strategy, which was clearly illustrated in walking time variation. Unfortunately, these detailed feed and ration data were not available and these effects could not be studied further.

The 24h patterns in this study present a clear diurnal rhythm per behavioral parameter. This pattern cannot be observed when utilizing sensor data on a daily scale. The main behavioral variations occurred in eating, standing and walking during the day and rumination and lying during the night. A nightly lying and rumination pattern described earlier seems consistent with our data [21]. We expect that cows in our study are able to present simultaneous lying and feeding behavior because these farms had neither overstocking in cubicles nor feeding places. Farms differed in the times of milking and fresh feed delivery, as well as in rational differences. These were not recorded. Differences between farms, as presented by AMS-C and CMS-P groups, were most clear in leg sensor data. Leg sensor data from cows in the CMS-P group showed more daily variations compared to cows in the AMS-C group. The daily patterns, however, are comparable between both groups. For instance, standing time has a peak in the morning and at the end of the afternoon in both groups. In the AMS-C group, cows have a voluntary milking system, while this is an obligatory moment in the CMS-P group.

Diurnal patterns in fully grazing systems are to our knowledge unknown. Circadian rhythms based on an indoor positioning system showed that deviations from this rhythm were useful to detect disease and estrus expression [23, 24]. This could imply that sensor data which monitors 24h patterns could give rise to specific algoritms for early disease detection in individual animals. Furthermore, our data provides a benchmark for sensor data to use in decision support in daily management such as feeding or monitoring welfare in lying and standing.

## Conclusions

This study presented the variability in time budget from the late dry period to the late lactation cycle. Time budgets differ between first, second and older cows, particularly eating time. As first parity cows showed different time budgets compared to older animals, these young animals might need specific management to better adapt to the milking herd. Time budgets of cows from different farm types were comparable. Finally, the dairy cows in this study showed a 24h pattern per behavioral parameter, indicating dairy cow behavior has a diurnal or circadian aspect.
